# Postoperative Olfaction Alteration Following Laparoscopic Bariatric Surgery

**DOI:** 10.3390/jcm10081704

**Published:** 2021-04-15

**Authors:** Magdalena Pisarska-Adamczyk, Piotr Tylec, Natalia Gajewska, Julia Wierzbicka, Krzysztof Przęczek, Piotr Małczak, Michał Wysocki, Michał Pędziwiatr, Mateusz Wierdak, Piotr Major

**Affiliations:** 2nd Department of General Surgery, Jagiellonian University Medical College, Jakubowskiego 2, 30-688 Kraków, Poland; magdalenapisarska@interia.pl (M.P.-A.); tylec.piotr@gmail.com (P.T.); natgajewska92@gmail.com (N.G.); Julia.weronika.wierzbicka@gmail.com (J.W.); krzysiek.przeczek@gmail.com (K.P.); piotr.malczak@doctoral.uj.edu.pl (P.M.); m.wysocki@doctoral.uj.edu.pl (M.W.); michal.pedziwiatr@uj.edu.pl (M.P.); piotr.major@uj.edu.pl (P.M.)

**Keywords:** bariatric surgery, olfaction change, early recovery after surgery protocol

## Abstract

Introduction: Bariatric surgery is an effective method of treatment for morbid obesity that is known to change nutritional habits. Proper nutrition has an impact on postoperative recovery and outcomes. Diet preferences depend on flavour and olfaction stimuli. Some studies show long-term changes in the sense of smell after bariatric surgery, but little research has addressed olfactory function shortly after surgery. Observations of olfactory changes that occur immediately after bariatric surgery may lead to improvements in postoperative care. Aim: The aim of this study is to investigate the impact of bariatric surgery on olfactory changes in the short postoperative period. Material and methods: This is a prospective study of patients undergoing laparoscopic sleeve gastrectomy (LSG) and laparoscopic Roux-en-Y gastric bypass (LRYGB) between April 2018 and December 2018. The control group consists of patients who underwent various non-oncological elective surgical procedures. Patients’ olfaction was tested qualitatively and quantitatively the day before and 24 h after surgery. Sniffin Sticks test consists of three subtests: odor threshold, discrimination, and identification. Results: The study enrolled 83 patients (LSG = 39; LRYGB = 14; control = 30). Mean scores in the threshold subtest differed significantly in the bariatric group, 9.3 ± 3.9 before the surgery and 8.2 ± 3.0 a day after the surgery (*p* = 0.032). There were no significant differences between scores from the discrimination subtest, identification subtest and the mean total scores in the bariatric group. There was no observed change in the intensity of the smell in the control group. Analyzing the results of patients undergoing LSG and LRYGB separately, we only showed significant differences in the case of LSG. Mean score from the 1st test (9.12 ± 3.97 vs. 7.75 ± 2.98; *p* = 0.0339) and mean total score (32.83 ± 5.45 vs. 30.67 ± 4.88; *p* = 0.0173) differ between repetitive measurements in LSG patients. Conclusions: Our study shows deterioration of odor threshold in the bariatric surgery group compared to the control group 24 h after surgery. However, this change occurred only in patients undergoing LSG.

## 1. Introduction

Laparoscopic bariatric surgery is a gold standard in the treatment of patients suffering from morbid obesity. Laparoscopic sleeve gastrectomy (LSG) and laparoscopic Roux-en-Y gastric bypass (LRYGB) are the two most commonly performed bariatric operations [1]. The main cause for obesity is excessive caloric intake caused by increased appetite and a deregulated neurohormonal food intake axis [2]. The latest scientific reports suggest a strict correlation between neurohormonal appetite axis hormones and olfaction [3]. Moreover, bariatric surgery not only causes a reduction in food intake, it also alters taste and smell preferences, which are connected to neurohormonal appetite regulation [4]. Observed long-term changes in olfaction after bariatric surgery may indicate the correlation between appetite regulation and sense of smell [5,6].

A significant amount of patients, after bariatric surgery, have a problem with consuming an appropriate amount of food to ensure a proper protein and energy balance, which may lead to a deterioration of their nutritional status, especially in terms of protein deficiencies [7,8]. Dietary recommendations in the postoperative period indicate the need to maintain an adequate nutritional status in order to reduce postoperative complications. Dietary restrictions aimed at reducing body weight should be introduced a few weeks later [8]. However, due to the gastric volume restriction and the observed changes in smell and taste sensation, many patients have difficulties with adequate nutrition in the first weeks after bariatric surgery [7].

Investigation of changes in olfaction that occur immediately after the procedure ought to improve our approach to nutrition and postoperative care. To date, the data on odor discrimination, identification and sensitivity to smells in this group of patients are sparse.

The aim of this study was to investigate the possible impact of bariatric surgery on olfactory processing (alteration in olfaction sensibility, sense discrimination, and smell identification) 24 h after surgery.

## 2. Material and Methods

### 2.1. Study Design

This is a prospective study to analyze patients that underwent laparoscopic bariatric surgery between April 2018 and December 2018. The control group underwent various elective non-oncological laparoscopic gastrointestinal procedures (cholecystectomy, Nissen fundoplication, Heller cardiomyotomy) in the study period. The study was designed according to the STROBE checklist for observational studies [9].

Patients were recruited in a university teaching hospital specialized in laparoscopic surgery. More than 200 bariatric procedures are performed annually at this unit. The criteria for bariatric surgery for treatment of morbid obesity follow the Polish Surgeons Society’s Guidelines of The Section of Metabolic and Bariatric Surgery, which include body mass index (BMI) ≥ 40 kg/m^2^ or BMI ≥ 35 kg/m^2^ with obesity comorbidities [10].

Adults who met the eligibility criteria for bariatric procedures (either LSG or LRYGB) were included in the study. We excluded patients with a history of revisional surgery, olfactory disorders (anosmia, hyposmia), diseases of laryngopharynx, and patients who received less than 1000 mL of fluids orally on the first postoperative day (POD).

Patients who received less than 1000 mL oral fluids on the 1st POD were unable to drink fluids due to postoperative nausea or vomiting. This is usually peripherally stimulated by mechanical problems, often affecting taste and smell, leading to decreased appetite.

To quantify the olfaction performance we used the Polish modified version of the Sniffin Sticks test, which is a standardized tool for the assessment of many aspects of olfaction [11,12].

The Sniffin Sticks test consists of three subtests: odor threshold, discrimination and identification tests.

#### 2.1.1. Test 1: Threshold

The threshold subtest has 16 levels of *n*-butanol concentration in a solvent. Samples containing *n*-butanol constituted test odorants. Those without *n*-butanol were odorless (blanks). The lowest *n*-butanol concentration was 0.00012%, and each subsequent level doubled in intensity. The highest *n*-butanol concentration was 4%. On each level, patients are asked to select the odorant out of three test samples, two of which are blanks. On level 1, patients were presented with the odorant with the lowest smell concentration. To ensure the correct result, the patient was tested twice if the correct sample was chosen.

#### 2.1.2. Test 2: Discrimination

The discrimination subtest also had sixteen levels. On each level, patients were presented with three odorants, out of which one had a different smell from the other two. On every level that patients correctly identified the outlier, they received one point.

#### 2.1.3. Test 3: Identification

In the identification subtest, patients were presented with sixteen odorant sticks. For each odorant, they were asked to select the correct smell descriptor from a list of four answers.

The maximum score for every subtest was sixteen points. Collectively, in the *Sniffin Sticks* test, patients could acquire forty-eight points. Odor samples were presented approximately two centimetres from the nostrils and for three seconds. Patients were tested preoperatively in the evening before bariatric procedure and in the evening of the first POD.

### 2.2. Endpoints

The primary endpoint was to evaluate the changes in any olfaction dimension after operation. The secondary endpoint was comparison of olfaction changes between patients undergoing different bariatric procedures (LSG vs LRYGB).

### 2.3. Treatment Protocols

Perioperative care was standardized according to Enhanced Recovery After Surgery (ERAS) protocol, which was introduced in our unit in 2012 [13,14]. According to the ERAS protocol, all smokers had to quit smoking or use nicotine replacement therapy 4–6 weeks before surgery. There was no need for a nasogastric tube in the postoperative period in any of the patients. All patients received a normal or diabetic diet the day before the surgery. From the day of surgery, patients were allowed a liquid diet in the form of oral nutritional support.

Surgical techniques for LSG and LRYGB were standardized and were described previously [15,16].

A standardized anesthetic protocol was used in all patients (bariatric and control group). Following preoxygenation, patients were given fentanyl (200–500 mcg *iv*). General anesthesia was induced with propofol (100–300 mg *iv*) and maintained with sevoflurane and fentanyl (100 mcg bolus every 30 min). Tracheal intubation was facilitated with rocuronium. Perioperatively, all patients received standard nausea and vomiting prophylaxis (ondansetron).

### 2.4. Ethical Approval

All procedures performed in studies involving human participants were in accordance with the ethical standards of the institutional and national research committee and with the 1964 Helsinki declaration and its later amendments or comparable ethical standards. The study was approved by the institutional review board on 20 April 2018 (decision number: 727.6120.4.2018). Written informed consent was obtained from all patients.

### 2.5. Statistical Analysis

All data were analysed with Statsoft STATISTICA v. 13. The results are presented as mean ± standard deviation (SD) or median with quartiles, when appropriate. The Shapiro–Wilk test was used to check for normal distribution of data. The *t*-test was used for normally distributed quantitative data. The Wilcoxon test was used for skewed variables. The results were considered statistically significant when the *p*-value was found to be less than 0.05. *p*-value correction was performed with respect to multiple comparisons.

## 3. Results

### 3.1. Baseline Characteristics of the Group

**Figure 1:**
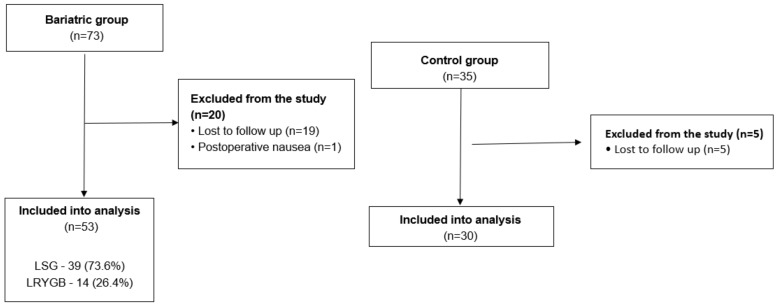
Patients flow through the study.

We included patients which were operated on in our unit between April and November 2018 (Figure 1).

#### 3.1.1. Bariatric Group

A total of 73 patients underwent bariatric procedures and were enrolled in our study. A total of 20 patients chose to discontinue their participation in the study. Ultimately, 53 patients met the inclusion criteria and completed both preoperative and postoperative sniff tests.

The mean age of the study participants was 46.6 ± 9.5 years. Mean BMI was 45.4 ± 5.1 kg/m^2^. A total of 22.6% of participants in bariatric group were ex-smokers, and this percentage was comparable between LSG and LRYGB patients.

#### 3.1.2. Control Group

A total of 35 patients were included in the control group. Thirty of them met the inclusion criteria and completed both tests. The mean age of the group was 48 ± 11 years. Mean BMI was 24.9 ± 9.1 kg/m^2^. A total of 26.7% of patients in this group were ex-smokers.

#### 3.1.3. Bariatric and Control Group Comparison

The Bariatric and Control groups were comparable in terms of sex, age and ASA scale. There was no statistically significant difference in percentage of ex-smokers between the control and bariatric groups. The Bariatric group had a higher BMI, prevalence (NAFLD) and pulmonary disease than the Control group. The differences in the prevalence of other comorbidities between the Bariatric and Control group were not statistically significant.

Characteristics of study groups are presented in Table 1.

### 3.2. Main Results

#### 3.2.1. Bariatric Group

A total of 39 (73.6%) patients underwent LSG, while 14 (26.4%) patients underwent LRYGB.

There were statistically significant differences between pre- and postoperative mean scores from the 1st test: 9.3 ± 3.9 vs. 8.2 ± 3.0 (*p* = 0.032). There were no statistical differences between scores from the 2nd test: 9.4 ± 2.9 vs. 8.9 ± 2.7 (*p* = 0.358). Mean results from the 3rd test also did not differ: 13.9 ± 1.6 preoperatively vs. 13.9 ± 1.9 after the surgery (*p* = 0.706). Moreover, we did not observe differences in mean total scores: 32.6 ± 5.5 and 31.0 ± 4.7 (*p* = 0.072).

Analysing the results of patients undergoing LSG and LRYGB separately, we only observed significant differences in the LSG subgroup. Mean score from the 1st test (9.12 ± 3.97 vs. 7.75 ± 2.98; *p* = 0.0339) and mean total score (32.83 ± 5.45 vs. 30.67 ± 4.88; *p* = 0.0173).

#### 3.2.2. Control Group

There were no statistically significant differences between pre- and postoperative mean scores from the 1st (8.9 ± 4.0 vs. 8.6 ± 3.6; *p* = 0.567), 2nd (9.5 ± 3.0 vs. 9.0 ± 3.2; *p* = 0.698) and 3rd (14.0 ± 1.6 vs. 13.9 ± 1.9; *p* = 0.541) tests. Moreover, we did not observe differences in mean total scores: 32.4 ± 5.5 and 31.5 ± 4.8 (*p* = 0.362).

The comparison of the bariatric and control group is presented in Table 2 and comparison of LSG and LRYGB patients in Table 3.

## 4. Discussion

The study showed that bariatric surgery, especially LSG, might alter olfaction in the postoperative period by reducing the perception of odor intensity. To our knowledge, this is the first study that analyzes this issue in bariatric surgery patients.

Almost all bariatric procedures interfere with stomach anatomy, especially the most frequently performed—LSG and LRYGB. By interfering with this section of the gastrointestinal tract, they affect the autonomic nervous system, and especially the vagus nerve [17]. In addition, through volume restriction, they accelerate the passage of gastrointestinal contents to the intestine [18,19]. The accelerated passage interferes with the secretion of neurohormones (ghrelin, glucagone-like peptide-1, peptide YY), whose action is also partially mediated by the autonomic nervous system [20,21]. This interference may be crucial for the observed changes in sense of smell. As many studies have shown, olfaction has a significant impact on the perception of food taste and may affect appetite.

Nutritional and bariatric surgery recommendations in the postoperative period indicate the need to maintain an adequate nutritional status in order to reduce postoperative complications [8,22,23,24]. Nevertheless, a significant number of patients after bariatric surgery have a problem with consuming an appropriate amount of food to ensure a proper protein and energy balance, which may lead to a deterioration in their nutritional status, especially in terms of protein deficiencies [7]. This could potentially increase postoperative morbidity and prolonged postoperative convalescence [25]. Due to postoperative changes related to the restriction of the volume of consumed food, as well as changes in the sensations of smell and taste, and limitations related to the volume and consistency of consumed food, it may be difficult for many patients to achieve an adequate nutritional supply in the postoperative period. Therefore, in our opinion, a better understanding of the changes in the perception of smell and taste can help optimize the taste and smell properties of recommended dietary supplements and introduce appropriate nutritional recommendations to improve the achievement of nutritional goals in the postoperative period.

There was no observed change in olfactory performance in the control group of patients undergoing various non-oncological elective surgery procedures for the upper gastrointestinal tract. Considering this, we suspect that the observed reduction in the sensitivity of the olfactory sense results from a bariatric procedure, rather than anaesthesia, injury or other factors. More studies are needed to fully understand the impact of the surgery on the change in olfaction immediately after operations.

Previous studies have assessed the change in olfactory senses several months after bariatric surgery. Reported data contradict the results of our study [5,6]. Observed differences may be related to the change in adaptation to signals mediated by vagus nerve. Studies have shown the stimulating effect of ghrelin secretion to increase the perception of odors [26]. This phenomenon is mediated by vagus nerve and is considered to be one of the mechanisms responsible for reducing the perception of odors in patients with morbid obesity in population-based studies [17]. Ghrelin is produced mainly by the fundus of the stomach, and that is why sleeve gastrectomy significantly reduces its secretion in the postoperative period. Unlike LRYGB, it does not damage the vagus nerve trunk and thus does not interfere so significantly with other functions regulated by the brain–gut axis. Other authors suggested that the increase in the perception of odor intensity may be associated with the temporary adaptation of the vagus nerve endings to a reduced amount of secreted ghrelin or with other non-vagal mechanisms [27]. Leptin, which is mainly secreted by the adipose tissue, has a significant impact on reducing the sensitivity of the olfactory centres [28,29]. In the case of postoperative body weight reduction, its total amount significantly decreases, and this may be the reason for the improvement in the perception of odour intensity observed in cited studies [27]. The proposed mechanism could be one of many possible explanations and further research is necessary to better understand the causes of the observed phenomenon.

As noted in the previous studies, the Sniffin Sticks test has some limitations [11]. Tested odors originate from outside the mouth and enter the olfactory bulb endings through the front nostrils. When food is consumed, the aroma and taste of food are analyzed in the brain together, giving the composed taste and smell impression of the consumed food. Both the aspiration route and the taste context can be important in the final assessment of the olfactory sensation [30].

However, this is the first study of obese patients who underwent bariatric surgery examining olfaction in the immediate postoperative period. Given the lack of research in this area, a correct prediction of the sample size was problematic; therefore, we based it on the mean difference results from previous long-term studies. Further research is needed to determine the real mechanisms behind the observed variation. We hope that the obtained data will be used to make better dietary recommendations, as well as provide a basis for further research to improve the tolerability of oral nutritional support used during postoperative recovery.

The study has several limitations. First of all, the analyzed group is relatively small, in addition, it comes from one geographical and genetic population, which may diminish the broad interpretation of our results. A small research group did not allow for a more accurate analysis of the impact of anthropometric parameters on the observed variability. Hence, the conducted study should be considered a pilot study. No long-term observations were made; therefore, the study cannot be directly compared to observations made in other studies [27]. One of the last limitations is the possibility of selection bias in both groups, because the Sniffin Sticks test is highly dependent on patient cooperation. We also did not explore the possible effects of anesthetics, but their use was comparable for all patients in both groups.

## 5. Conclusions

Our study showed that the olfactory threshold may deteriorate within 24 h after bariatric surgery. This change did not occur in the control group of patients undergoing other gastrointestinal procedures. Furthermore, the olfactory performance was altered only in the LSG subgroup of bariatric patients.

## Figures and Tables

**Table 1:** Demographic analysis of patient groups.

Parameter	Bariatric Group	Control Group	*p* Value
Number of patients, n	53	30	-
Females, *n* (%)	28 (52.8%)	17 (56.7%)	0.736
Males, *n* (%)	25 (47.2%)	13 (43.3%)	
Mean age, years ± SD	46.6 ± 9.5	48.1 ± 11.2	0.519
Mean BMI, kg/m^2^ ± SD	45.4 ± 5.1	24.9 ± 9.1	<0.001
Mean Maximal BMI, kg/m^2^ ± SD	48.4 ± 6.0	-	-
Type of surgery, *n* (%)	LSG—39 (73.6%) LRYGB—14 (26.4%)	Cholecystectomy—23 (76.7%) Nissen fundoplication—4 (13.3%) Heller cardiomyotomy—3 (10%)	-
Ex-smokers, *n* (%)	12 (22.6%)	8 (26.7%)	0.680
ASA 2, *n* (%)	40 (75.5%)	26 (86.7%)	0.225
ASA 3, *n* (%)	13 (24.5%)	4 (13.3%)	
Hypertension, *n* (%)	37 (69.8%)	22 (73.3%)	0.734
Diabetes, *n* (%)	21 (39.6%)	5 (16.7%)	0.055
Dyslipidemia, *n* (%)	12 (22.6%)	3 (10%)	0.254
NAFLD, *n* (%)	14 (26.4%)	1 (3.3%)	0.020
Pulmonary disease, *n* (%)	19 (35.9%)	3 (10%)	0.021
Mean operative time, min ± SD	93 ± 32	89 ± 36	0.603

BMI—Body Mass Index, ASA scale—American Society of Anaesthesiology scale, NAFLD—Non-Alcoholic Fatty Liver Disease, LSG—Laparoscopic Sleeve Gasrectomy, LRYGB—Laparoscopic Roux-en-Y gastric bypass.

**Table 2:** Test results in bariatric and control groups.

Parameter	Preoperative Test	Postoperative Test	*p* Value
Bariatric group			
Median Subjective olfactory evaluation, points (IQR)	2 (1–2)	2 (1–2)	0.3882
Mean Test 1, points ± SD	9.33 ± 3.88	8.19 ± 3.02	0.0320
Mean Test 2, points ± SD	9.40 ± 2.94	8.92 ± 2.65	0.3588
Mean Test 3, points ± SD	13.91 ± 1.63	13.91 ± 1.88	0.7063
Mean total, points ± SD	32.63 ± 5.47	31.02 ± 4.72	0.0716
Control group			
Median Subjective olfactory evaluation, points (IQR)	2 (1–2)	2 (1–2)	0.4722
Mean Test 1, points ± SD	8.91 ± 3.97	8.64 ± 3.59	0.5670
Mean Test 2, points ± SD	9.52 ± 2.99	9.01 ± 3.15	0.6984
Mean Test 3, points ± SD	13.98 ± 1.59	13.88 ± 1.89	0.5409
Mean total, points ± SD	32.39± 5.47	31.53 ± 4.81	0.3621

**Table 3:** Test results in LRYGB and LSG patients.

Parameter	Preoperative Test	Postoperative Test	*p* Value
LRYGB			
Median Subjective olfactory evaluation, points (IQR)	2 (1–2)	2 (1–2)	0.3613
Mean Test 1, points ± SD	9.85 ± 3.82	9.44 ± 3.03	0.6496
Mean Test 2, points ± SD	8.62 ± 3.28	8.39 ± 3.2	0.8613
Mean Test 3, points ± SD	13.85 ± 1.68	14.15 ± 1.68	0.3627
Mean total, points ± SD	32.31 ± 5.84	31.98 ± 4.41	0.8613
LSG			
Median Subjective olfactory evaluation, points (IQR)	2 (1–2)	2 (1–2)	0.9326
Mean Test 1, points ± SD	9.12 ± 3.97	7.75 ± 2.98	0.0339
Mean Test 2, points ± SD	9.74 ± 2.79	3.05 ± 2.48	0.197
Mean Test 3, points ± SD	13.97 ± 1.63	13.87 ± 1.96	0.9142
Mean total, points ± SD	32.83 ± 5.45	30.67 ± 4.88	0.0173

## Data Availability

The data presented in this study are available on request from the corresponding author.
